# Simultaneous Effects of Light Intensity and Phosphorus Supply on the Sterol Content of Phytoplankton

**DOI:** 10.1371/journal.pone.0015828

**Published:** 2010-12-31

**Authors:** Maike Piepho, Dominik Martin-Creuzburg, Alexander Wacker

**Affiliations:** 1 Institute of Biochemistry and Biology, Theoretical Aquatic Ecology, University of Potsdam, Potsdam, Germany; 2 Limnological Institute, University of Constance, Konstanz, Germany; Purdue University, United States of America

## Abstract

Sterol profiles of microalgae and their change with environmental conditions are of great interest in ecological food web research and taxonomic studies alike. Here, we investigated effects of light intensity and phosphorus supply on the sterol content of phytoplankton and assessed potential interactive effects of these important environmental factors on the sterol composition of algae. We identified sterol contents of four common phytoplankton genera, *Scenedesmus*, *Chlamydomonas*, *Cryptomonas* and *Cyclotella*, and analysed the change in sterol content with varying light intensities in both a high-phosphorus and a low-phosphorus approach. Sterol contents increased significantly with increasing light in three out of four species. Phosphorus-limitation reversed the change of sterol content with light intensity, i.e., sterol content decreased with increasing light at low phosphorus supply. Generally sterol contents were lower in low-phosphorus cultures. In conclusion, both light and phosphorus conditions strongly affect the sterol composition of algae and hence should be considered in ecological and taxonomic studies investigating the biochemical composition of algae. Data suggest a possible sterol limitation of growth and reproduction of herbivorous crustacean zooplankton during summer when high light intensities and low phosphorus supply decrease sterol contents of algae.

## Introduction

Sterols are important structural and functional molecules in eukaryotic cells. They are involved in the regulation of membrane fluidity, signal transduction and modulation of the activity of membrane-bound enzymes [1]. In animals and higher plants, sterols are precursors of steroid hormones and involved in the synthesis of a variety of secondary metabolites [2]. In contrast to animals, which predominantly contain cholesterol, plants and algae contain a great diversity of different phytosterols. These sterols differ in their number and position of double bonds in the ring structure of the tetracyclic molecule and in the structure of the side chain. Until now it is unclear if this variety in sterol composition entails an advantage for plants and if individual sterols play specific roles in plant cell metabolism [2]. Sterols are able to regulate membrane permeability and fluidity but in higher plants e.g. stigmasterol was found to be less efficient than sitosterol and 24-methylcholesterol [3]. Grandmougin-Ferjani et al. [4] revealed that cholesterol and stigmasterol are able to stimulate the H^+^ pump of the ATPase in higher plant plasma membranes. These examples suggest that specific sterols may have particular functions in plant cells apart from their general role as structural components in cell membranes.

The sterol composition of algae is of great interest for several reasons. Microalgae are an important source of sterols for higher trophic levels in the aquatic food web. Although most eukaryotic organisms are able to synthesize sterols *de novo*, presumably all arthropods depend on ingesting sterols with their diet [5]. The main sterol in arthropods is cholesterol, which is characterized by a double bond at position Δ^5^ in the ring structure. Most phytosterols that are ingested with food are more or less efficiently converted into this molecule [6], [7]. However, not all of the great number of phytosterols in algae are suitable as cholesterol precursors in crustacean zooplankton [8]–[10]. For example, sterols with double bonds at positions Δ^0^, Δ^8^ or Δ^4^ apparently cannot be converted into cholesterol. Food quality in terms of sterols therefore greatly depends on the sterol composition of algal species in zooplankton diet [7], [8].

Sterol compositions are also used in taxonomic studies [11]. Sterol profiles might serve as chemotaxonomic markers to distinguish species or to identify sources of organic matter in sediments. However, there is still not enough information about the occurrence of sterols in some taxonomic groups. For all these reasons an understanding of differences in sterol composition between phytoplankton species and variations with environmental conditions, such as light intensity, is important. In plants and algae, research has focused on the diverse sterol composition of different species [1], [2], [12] but there are very few studies that deal with the influence of culture conditions on the sterol content or composition of algae. Some studies suggest that the sterol content of algae changes with environmental conditions, such as nutrient supply or light intensity [13], [14]. As yet, however, simultaneous effects of nutrient and light availability have not been considered.

Therefore, we tested the influence of different light intensities on the sterol content and composition of phytoplankton species under both high-P and low-P conditions. We chose four phytoplankton genera (*Scenedesmus*, *Chlamydomonas*, *Cryptomonas* and *Cyclotella*) that are widespread in freshwater lakes and so are important food components for zooplankton. High light intensities and low phosphorus availability are common in many lakes during summer. By analysing both conditions simultaneously we were able to detect interactive effects of the two factors light intensity and phosphorus supply, i.e. phosphorus supply influenced the reaction of algal sterol contents to varying light intensities.

## Methods

The two Chlorophyceae *Scenedesmus quadricauda* (Turpin) Brébisson and *Chlamydomonas globosa* J.W. Snow, the Cryptophyceae *Cryptomonas ovata* Ehrenberg (all three species from the culture collection of the Limnological Institute, University of Constance), and the Mediophyceae (Bacillariophyta) *Cyclotella meneghiniana* Kützing (collection of algal cultures, Göttingen, SAG 1020-1a) were cultivated in WC-medium [15] with one high-P (50 µM P) and one low-P-medium per species and continuous light. To achieve light intensities of 30, 60, 140, 230, and 490 µmol photons m^−2^s^−1^, a climate chamber (20°C) was divided into compartments. Each was shaded differently with foil (Lee Colour correction filters, Neutral density, Lee Filters, Hampshire, England). The low-P-medium was 1 µM P for *Scenedesmus*, 5 µM P for *Cryptomonas* and *Chlamydomonas* and 10 µM P for *Cyclotella* because of species-specific differences in their optimum phosphate requirements. We had two replicates per light compartment of each treatment. Carbon deficiency was avoided by aerating the media with sterile-filtered air. We used semi-continuous culture conditions, i.e. diluted the cultures every day at the same time (dilution rate: 0.2 d^−1^). The cultures were grown until the growth rate remained constant. Then the experiment was continued for three more weeks to ensure that all cells of a culture have the same cell division rate and to achieve photoacclimation of the cultures. At the end of the experiment light intensities within all cultures were determined separately. Slightly different growth of the replicates of each species in the same light compartment resulted in differing cell densities and therefore in slightly differing light conditions due to shading. We therefore decided to deal with all cultures as individual levels in a continuous light gradient instead of referring to the cultures in the same light compartment as replicates.

Samples for sterol determination were obtained by filtering 0.5–1 mg carbon on glass fiber filters (25 mm GF/F, Whatman). Filters were stored at −23°C under nitrogen atmosphere in glass tubes with Teflon seals after adding 7 ml of dichlormethane-methanol (2∶1 v/v) for the extraction of lipids. Before further analysis a defined concentration of 5α-cholestan (Sigma-Aldrich) was added as internal standard. After extraction of lipids, identification and quantification of sterols was done by gas chromatography (6890N Network GC System, Agilent Technologies) according to Wacker and Martin-Creuzburg [16] but with the following configuration: 1 µl of sample was injected in the split mode (5∶1), vaporized in the injector at 350°C and mixed with the carrier gas (helium). Sterols were separated by a polysiloxane column (Agilent technologies J&W HP-5, 30 m×0.32 mm×0.25 µm) and a temperature gradient (150°C for 1 min, 15°C min^−1^ until 280°C, 2°C min^−1^ until 308°C, 10°C min^−1^ until 320°C). Detection was done using a flame ionisation detector (FID) at 350°C. Quantification of sterols was done by means of added internal standards and by using multipoint standard calibration curves determined for each sterol from mixtures of known composition. Sterols were identified via known retention times of reference substances (Sigma-Aldrich) and their mass spectra, which were recorded with a gas chromatograph-mass spectrometer (Finnigan MAT GCQ) equipped with a fused-silica capillary column (DB-5MS, Agilent; GC configurations as described in Martin-Creuzburg et al. [17]). Sterols were analysed in their free form and as their trimethylsilyl derivatives, which were prepared by incubating 20 ml of iso-hexane sterol extract with 10 ml of N,O-bis(trimethylsilyl)trifluoroacetamide including 1 per cent trimethylchlorosilane for 1 hour at room temperature. Spectra were recorded between 50 and 600 amu in the EI ionization mode.

For determination of particulate organic carbon (POC) ca. 0.25 µg carbon were filtered on precombusted glass fiber filters (25 mm GF/F, Whatman) and analysed using a High-TOC Analyser (Elementar Analysensysteme GmbH, Hanau, Germany).

Sterol contents of algae were expressed on a per carbon basis and differences between algae were analysed by Analyses of Covariance (ANCOVA) with light intensity as continuous variable and medium phosphorus concentration as factor. The statistical test resulted in four possibilities to describe the reaction of sterol contents to experimental conditions: (1) There is a significant reaction to light but not to phosphorus concentrations, i.e. no difference between the high-P and low-P treatments; (2) there is a significant difference between the high-P and low-P treatments but no reaction to light; (3) both light and phosphorus supply have a significant influence on the contents of sterols; (4) the two factors interact, i.e. reaction to light differs depending on phosphorus supply. To meet uniformity of variance assumptions, light intensities were log_e_ transformed. All statistical calculations were carried out using the statistical software package R version 2.6.0, which is under general public licence (R Development Core Team, 2007).

## Results

The four freshwater species *Scenedesmus quadricauda* (Chlorophyceae), *Chlamydomonas globosa* (Chlorophyceae), *Cryptomonas ovata* (Cryptophyceae) and *Cyclotella meneghiniana* (Mediophyceae) possess different sterol compositions and also differ in their total sterol content. The highest sterol content (10 µg mgC^−1^ on average) was found in *S. quadricauda*. *C. meneghiniana* and *C. ovata* had an average sterol content of 7–8 µg mgC^−1^. In *C. globosa* the lowest sterol content was observed (4 µg mgC^−1^ on average).

**Figure 1:**
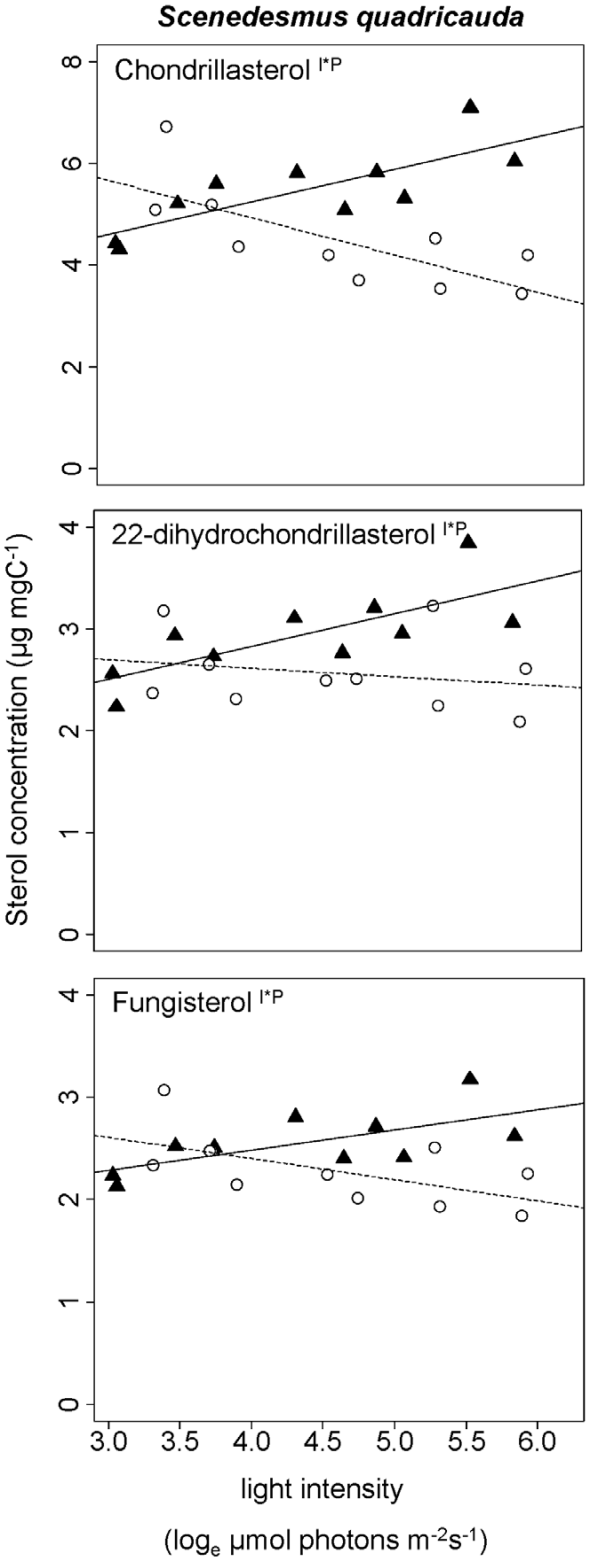
Change in sterol content with light intensity in *Scenedesmus quadricauda*. (▴, straight line) High-P treatments. (○, dashed line) Low-P treatments. Each data point represents a single culture. Superscripts indicate statistically significant effects of light intensity (l), phosphorus supply (P), or interaction of these two factors (l*P). A regression line was added if one of the effects was significant.

In *S. quadricauda*, fungisterol (IUPAC name: 5α-ergost-7-en-3β-ol), chondrillasterol ((22E)-5α-poriferasta-7,22-dien-3β-ol), and 22-dihydrochondrillasterol (5α-poriferast-7-en-3β-ol) were detected (Fig. 1). Statistical analyses of the effects of light and phosphorus supply on the content of each of these three sterols in *S. quadricauda* revealed significant interactions (Table 1), i.e. in the high-P treatment the sterol content increased with light intensity but decreased in the low-P treatment. This effect was most pronounced for chondrillasterol, whose content increased by 50% in the high-P treatment but decreased by the same factor in the low-P treatment. The total sterol content in *S. quadricauda* increased from 9 to 13 µg mgC^−1^ in the high-P treatment and decreased from 11 to 8 µg mgC^−1^ in the low-P treatment.

**Table 1:** P-values of ANCOVA for changes in sterol contents of four phytoplankton species, depending on the continuous variable light intensity (l, log_e_-transformed) and the factor medium phosphorus concentration (P).

	*Scenedesmus quadricauda*			*Chlamydomonas globosa*			*Cryptomonas ovata*			*Cyclotella meneghiniana*		
	l	P	l*P	l	P	l*P	l	P	l*P	l	P	l*P
Brassicasterol	-	-	-	-	-	-	0.74	0.82	0.93	-	-	-
Ergosterol	-	-	-	0.41	**<0.001**	0.71	-	-	-	-	-	-
24-methylenecholesterol	-	-	-	-	-	-	-	-	-	0.07	**<0.05**	**<0.01**
22-dihydrobrassicasterol	-	-	-	-	-	-	-	-	-	**<0.01**	0.35	**<0.01**
Stigmasterol	-	-	-	-	-	-	0.85	**<0.05**	0.53	-	-	-
Fungisterol	0.74	**<0.05**	**<0.01**	**<0.01**	**<0.001**	**<0.001**	-	-	-	-	-	-
Chondrillasterol	0.47	**<0.01**	**<0.001**	-	-	-	-	-	-	-	-	-
22-dihydrochondrillasterol	0.27	**<0.05**	**<0.05**	-	-	-	-	-	-	-	-	-

**Figure 2:**
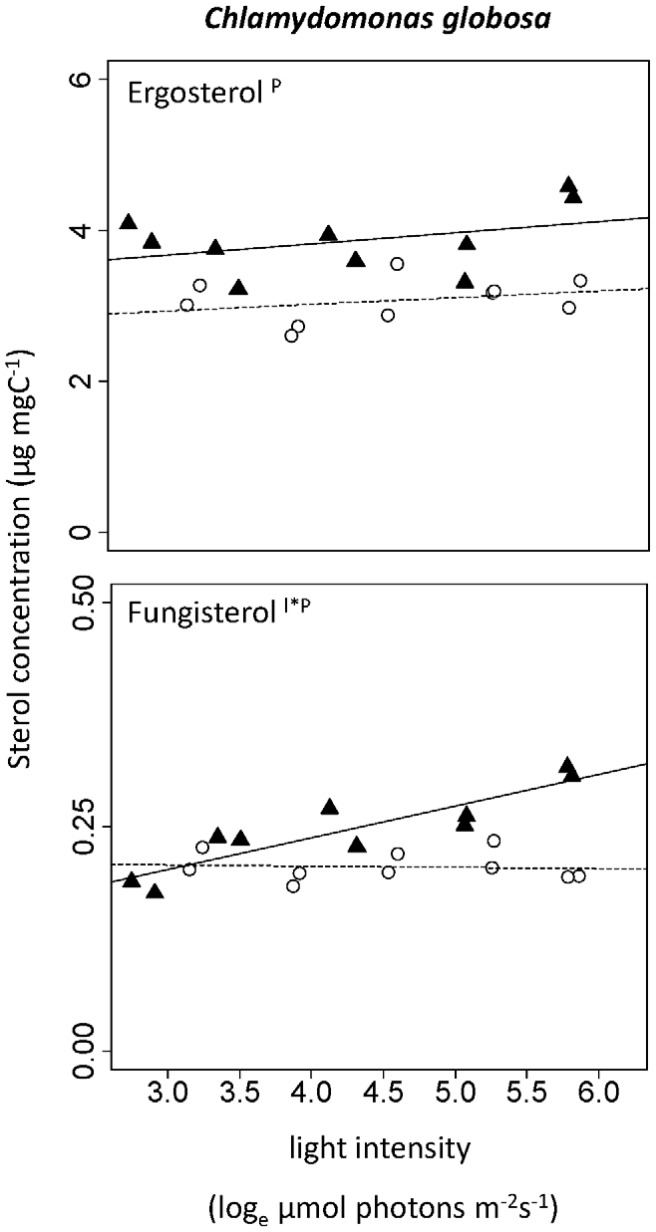
Change in sterol content with light intensity in *Chlamydomonas globosa.* (▴, straight line) High-P treatments. (○, dashed line) Low-P treatments. Superscripts indicate statistically significant effects of light intensity (l), phosphorus supply (P), or interaction of these two factors (l*P). A regression line was added if one of the effects was significant.

The only sterol we found in more than one of the analysed species was fungisterol, which was abundant in the two green algae *S. quadricauda* and *C. globosa*. In *C. globosa*, the same interaction as in *S. quadricauda* was observed for fungisterol (Table 1). Though this sterol was present in very low amounts, fungisterol doubled in content in the high-P treatment whereas the content decreased slightly in the low-P treatment (Fig. 2). Besides fungisterol we also found ergosterol ((22E)-ergosta-5,7,22-trien-3β-ol) in *C. globosa*, which in fact was present in much higher amounts than fungisterol. Ergosterol content did not change with light but increased with phosphorus availability, i.e. in the high-P treatment (Table 1, Fig. 2). The last-mentioned effect was also significant in the total sterol content of *C. globosa*: in the high-P treatment the content was 4 µg mgC^−1^ but in the low-P treatment only 3 µg mgC^−1^.

**Figure 3:**
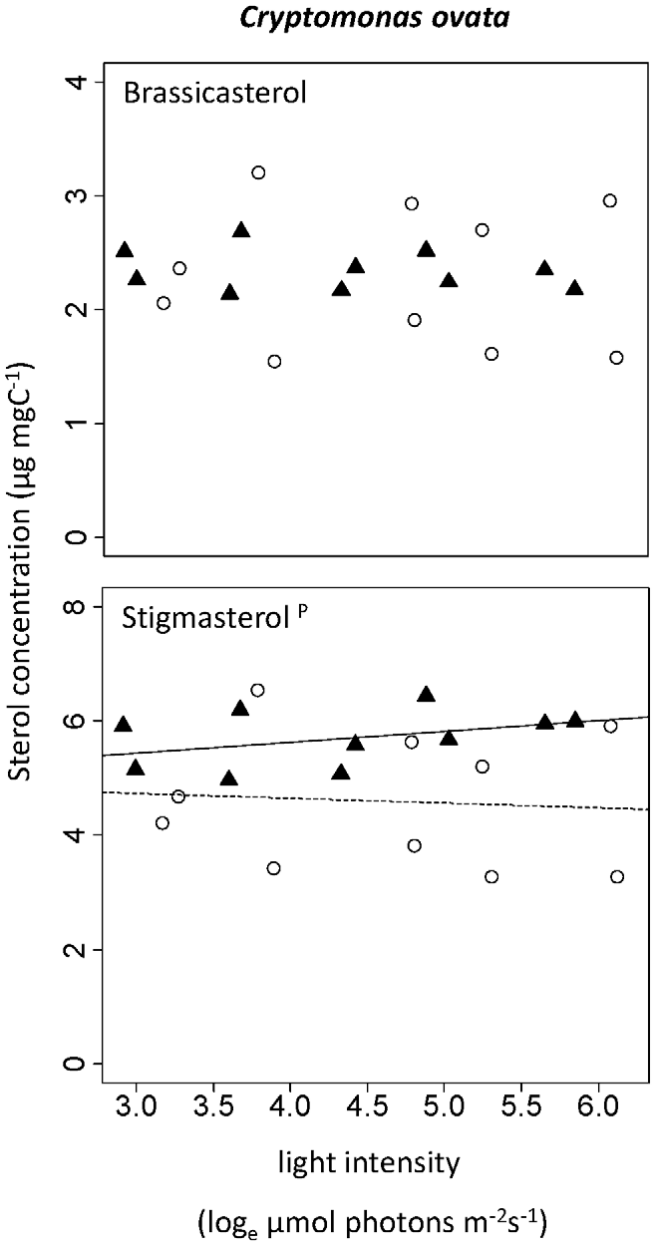
Change in sterol content with light intensity in *Cryptomonas ovata*. (▴, straight line) High-P treatments. (○, dashed line) Low-P treatments. Superscripts indicate statistically significant effects of light intensity (l), phosphorus supply (P), or interaction of these two factors (l*P). A regression line was added if one of the effects was significant.

In *C. ovata*, brassicasterol ((22E)-ergosta-5,22-dien-3β-ol) and stigmasterol ((22E)-stigmasta-5,22-dien-3β-ol) were the principal sterols (Fig. 3). Whereas the content of the first did not change with any of the experimental conditions, the content of the latter was higher in the high-P than in the low-P treatment (Table 1), but did not change with light intensity. No significant change in sterol content was seen when we examined the sum of sterols in *C. ovata*.

**Figure 4:**
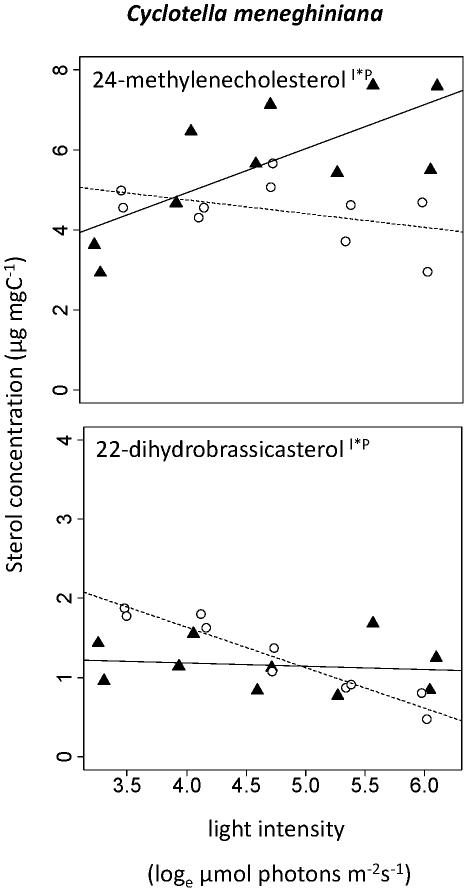
Change in sterol content with light intensity in *Cyclotella meneghiniana*. (▴, straight line) High-P treatments. (○, dashed line) Low-P treatments. Superscripts indicate statistically significant effects of light intensity (l), phosphorus supply (P), or interaction of these two factors (l*P). A regression line was added if one of the effects was significant.

24-methylenecholesterol (ergosta-5,24(24^1^)-dien-3β-ol) and 22-dihydrobrassicasterol (ergost-5-en-3β-ol) were detected in *C. meneghiniana* (see discussion for details about identification of sterols). Consistent with the results obtained for *S. quadricauda* we found interactive effects of the two factors light and phosphorus supply on both sterols in *C. meneghiniana* (Table 1). 24-methylenecholesterol content increased about 2-fold with light in the high-P treatment whereas it decreased 1.5-fold in the low-P treatment (Fig. 4). The content of 22-dihydrobrassicasterol did not change with light availability in the high-P treatment but decreased with light in the low-P culture (Fig. 4). The total sterol content increased from 5 to 8 µg mgC^−1^ in the high-P treatment but decreased from 7 to 5 µg mgC^−1^ in the low-P treatment.

## Discussion

We observed significant changes of sterol contents with light intensity and phosphorus supply. Interestingly, sterols of *S. quadricauda* and *C. meneghiniana* increased with light intensity in the high-P treatment, but decreased with light intensity in the low-P treatment. In general, sterol contents tended to be lower in low-P algae, at least at medium to high light intensities. Where we observed interactions, we found higher sterol contents at low light in the low-P cultures than in the high-P treatments.

To date, there have not been many studies on the effects of changing environmental conditions on the sterol content of algae. In accordance with our results, Gordillo et al. [13] observed an increase of sterols with increasing light intensities in the halotolerant green alga *Dunaliella*. In contrast, Parrish et al. [18] found the sterols in the marine dinoflagellate *Gymnodinium* to be more abundant in low light. Since in the latter study the algae were grown in batch cultures for several days a P-limitation cannot be ruled out. If this was the case, the results of Parrish et al. [18] would be consistent with our results for low-P *S. quadricauda* and *C. meneghiniana*.

There are some possible explanations for the observed changes in sterol contents. A reason for increasing sterol contents with increasing light could be the biosynthesis pathways of different algae. Sterols can be synthesized in two possible ways: (1) The MVA (mevalonate) pathway, where the sterol precursor isopentenyl diphosphate (IPP) is synthesised in the cytosol from acetyl-CoA via MVA and (2) the MEP (methylerythritol) pathway in which IPP is synthesised in the chloroplast from glyceraldehyde-3-phosphate and pyruvate. Whereas green algae seem to use only the MEP pathway for sterol synthesis, sterols of other microalgae were found to be synthesised via the MVA pathway [1], [19]. To our knowledge, the biosynthesis pathway of sterols in *Cryptomonas* is not known. In most algal classes both pathways, MEP and MVA, seem to be involved in isoprenoid biosynthesis. However, studies using isotopic labelling techniques [19], [20] showed that in general only one of the two pathways leads to sterols as end products. For example, in one Rhodophyte and one Chrysophyte, chloroplast isoprenoids, such as phytol and β-carotene, were synthesized via MEP-pathway, but sterols derived from MVA-pathway [19]. In some diatoms the MEP-pathway was found to be responsible for sterol synthesis [20], but apparently in different diatom species sterol formation via MVA-pathway also is possible [1]. Therefore, evolutionary pathways seem not to correlate with metabolic pathways of sterol formation, since there are also differences between algae of the same phylum (diatoms). Disch et al. [19] hypothesized that the MEP pathway is related to photosynthesis as it is linked with the chloroplast. This could explain the increase of sterols with increasing light in the green alga *S. quadricauda* and the diatom *C. meneghiniana*. Higher light intensities stimulate photosynthesis and possibly the production of sterols via the MEP pathway.

To explain the interacting effects of light and phosphorus on the sterol contents of the algae, we propose that at high light intensities the increased carbon fixation also allows an increased synthesis of sterols. On the other hand, sterol production seems to be constrained by a low phosphorus supply, as indicated by the lower sterol content in at least some low-P treatments. Therefore, low-P algae might not be able to synthesize increasing amounts of sterols with increasing light intensities. Since we analysed the sterol contents on a per carbon basis, a carbon accumulation (e.g. storage molecules like carbohydrates or lipids), as it is often found in P-limited cells [21], might also explain the lower sterol content in the low-P treatments of our experiment. Moreover, an increase of carbon storage can also be observed under high light conditions [21]. In P-limited algae, a further increased carbon accumulation with increasing light intensities consequently would result in a decrease of sterols per carbon and could therefore explain the observed decrease in sterol contents. A more functional explanation could be that a low P-content in the cell constrains the building of plasma membranes, which contain great amounts of phospholipids, and therefore indirectly alter sterol content, since sterols are found predominantly in these membranes. On the other hand enzymes involved in sterol synthesis in plants are associated with membranes [2]. As a result, phosphorus supply might indirectly limit sterol synthesis by reducing the amount of membranes and so the enzymes needed for sterol synthesis.

However, not all of the analysed sterol contents showed the same reaction to light intensity and phosphorus supply. The content of ergosterol in *C. globosa* and the content of stigmasterol in *C. ovata* did not change with light intensity but both were higher in the high-P than in the low-P treatment. Brassicasterol content in *C. ovata*, on the other hand, did not change with any of the experimental conditions. The reason for these specific reactions is not clear. The sterols might have different, yet unknown, functions in the cell that result in different reactions to environmental conditions. Furthermore, the above mentioned light and nutrient dependent carbon accumulation is realized in varying degrees in different species. For example, in contrast to the other species, in *C. ovata* no accumulation of storage lipids was found at high light intensities or low phosphorus supply (unpubl. data). If changes in sterol content per carbon can partly be explained by changes in carbon content, species specific differences in accumulation of carbon might lead to divergent reactions of sterol contents to the experimental conditions.

Our study revealed great differences between the sterol profiles of the analysed algae. It is known that for example the fatty acid profiles of algae belonging to the same class are quite similar (e.g., [22]). In contrast, the sterols of the two Chlorophyceae *S. quadricauda* and *C. globosa* clearly differed. The sterol profile of *S. quadricauda* was characterised by fungisterol, chondrillasterol and 22-dihydrochondrillasterol, three sterols characterised by a double bond at position Δ^7^, which have been reported previously to occur in *Scenedesmus obliquus* [23]. In contrast, the second green alga *C. globosa* contained fungisterol and considerable amounts of the Δ^5,7,22^ sterol ergosterol. Though the presence of ergosterol is frequently discussed as a characteristic feature of fungi [1], [24], the occurence of ergosterol in green algae has been reported previously (e.g., [25]). The two Δ^5,22^ sterols stigmasterol and brassicasterol were the principal sterols detected in *C. ovata*. It has been reported that cryptophycean algae such as *Cryptomonas* and *Rhodomonas* contain epibrassicasterol, the 24α-epimer of brassicasterol [26], [27]. Though we did not determine the side-chain stereochemistry at C-24, the presence of epibrassicasterol rather than brassicasterol in *C. ovata* was also assumed (cf. [28]). 24-Methylenecholesterol (Δ^5,24^) and a 24-methylsterol (Δ^5^) were detected in *C. meneghiniana*, which confirms previous studies [29]. According to Gladu et al. [29], the C-24 methyl group of the 24-methylsterol present in *Cyclotella cryptica* is β-oriented, indicating the presence of 22-dihydrobrassicasterol (ergost-5-en-3β-ol) rather than its 24α-epimer campesterol (campest-5-en-3β-ol).

Comparison of sterol contents of the four algae species suggests differences in their food quality for crustacean zooplankton. Martin-Creuzburg & von Elert [8] suggested that dietary phytosterols with double bonds at specific positions within the ring system of the sterol, namely Δ^5^ and Δ^5,7^ sterols, can be converted into cholesterol in the metabolism of the freshwater key herbivore *Daphnia*, whereas Δ^0^, Δ^8^ and Δ^4^ sterols cannot be converted and thus are not suitable to meet the sterol requirements for *Daphnia* growth and reproduction. *C. ovata* and *C*. *meneghiniana* both contain exclusively sterols with a double bond at position Δ^5^ in the ring system, which presumably can effectively be converted into cholesterol by *Daphnia*. The same applies to the Δ^5,7,22^ sterol ergosterol in *C*. *globosa*. Martin-Creuzburg & von Elert [8] suggested that sterols unsaturated at position Δ^7^ instead of position Δ^5^ in the ring system can be converted into cholesterol. However, they supported growth and reproduction of *Daphnia* to a significantly lower extent than cholesterol or the other Δ^5^ and Δ^5,7^ sterols tested in their study. All three sterols we found in *S. quadricauda* as well as fungisterol in *C. globosa* possess double bonds at position Δ^7^. This implies that *S. quadricauda* is a less valuable food for *Daphnia* in respect to sterols than the other investigated algae.

High light intensities and phosphorus-limited conditions are frequently observed in oligo- to mesotrophic lakes during summer. Our data suggests that at these summer conditions a sterol-limitation of the herbivore crustacean *Daphnia* is possible. The saturation threshold of dietary cholesterol for growth of *Daphnia* was described in different studies to be in the range of 5-6 µg mgC^−1^ [17], [23] or 7–9 µg mgC^−1^ [30], depending on the method of supplementation, temperature or presence of other food components such as polyunsaturated fatty acids [31]. Assuming that the total amount of phytosterols is converted into cholesterol, the here shown species do not reach these contents under all conditions. The species with the lowest sterol content, *C. globosa*, contained total sterol contents of <5 µg mgC^−1^, which is clearly below all suggested saturation levels. In *C. meneghiniana* high light intensities together with low P-supply led to total sterol contents <5 µg mgC^−1^. Adopting the higher threshold for sterol limited growth for *Daphnia* of 7–9 µg mgC^−1^, total sterol content of *C. ovata* under all conditions (7–8 µg mgC^−1^) and of *S. quadricauda* exposed to high light and low-P (8 µg mgC^−1^) were in the range of the threshold.

On the other hand, some phytosterols might be converted more efficiently into cholesterol than others. According to the study of Martin-Creuzburg and von Elert [8] the species with the highest total amount of sterols, *S. quadricauda*, is also the species that contains the least valuable sterols for crustacean zooplankton. Consequently, sterol limitation of *Daphnia* growth and reproduction is still possible even though total sterol content of the dietary phytoplankton species is above the mentioned threshold.

Sperfeld and Wacker [30] furthermore stated that the demand of *Daphnia* for sterols increases with increasing temperature. A sterol limitation of *Daphnia* in the field could therefore be increased in summer by high light intensities and low phosphorus supply, which reduce sterol contents in algae, and at the same time by higher temperatures, which increase the demand of *Daphnia* for sterols.

### Conclusion

With our investigation on the change in sterol content of four species of algae with increasing light intensity, simultaneously in a high-phosphorus and a low-phosphorus approach, we demonstrated that phosphorus supply is of great importance when discussing the effect of light intensity on the sterol content. In three out of four species we found contrary reactions to light depending on phosphorus supply. The diverse sterol composition of the investigated algae indicated possible variations in the availability of adequate cholesterol precursors for crustacean zooplankton. Furthermore, the data suggest that a sterol limitation of consumers is most likely in lakes during summer conditions.
